# A Unified Hybrid Model for Cardiovascular Risk Prediction: Merging Statistical, Kernel‐Based and Neural Approaches

**DOI:** 10.1111/jcmm.70797

**Published:** 2025-08-28

**Authors:** Mudassir Khan, Rupali A. Mahajan, Nithya Rekha Sivakumar, Monali Gulhane, Nitin Rakesh, Rajesh Dey, Md. Salah Uddin, Shakila Basheer

**Affiliations:** ^1^ Department of Computer Science, College of Computer Science, Applied College Tanumah King Khalid University Abha Saudi Arabia; ^2^ Vishwakarma Institute of Technology Pune India; ^3^ Department of Computer Sciences, College of Computer and Information Science Princess Nourah bint Abdulrahman University Riyadh Saudi Arabia; ^4^ Department of CSE Symbiosis Institute of Technology, Nagpur Campus, Symbiosis International (Deemed University) Pune India; ^5^ Gopal Narayan Singh University Jamuhar Bihar India; ^6^ Department of Multimedia and Creative Technology Daffodil International University Dhaka Bangladesh; ^7^ Department of Information Systems, College of Computer and Information Science Princess Nourah bint Abdulrahman University Riyadh Saudi Arabia

**Keywords:** cardiovascular risk prediction, hybrid machine learning, logistic regression, neural networks, predictive analytics, support vector machines

## Abstract

Cardiovascular diseases (CVDs) are still the leading cause of death in the worldwide. Traditional machine learning models often have difficulty in determine how to capture the complex links between disease risk factors and disease occurrence. This article discusses a hybrid machine learning approach for cardiovascular risk prediction (HMLCRP) to address this problem. This approach combines logistic regression (LR), support vector machines (SVMs) and neural networks (NNs) to make predictions more correct and reliable. The proposed model looks at important coronary heart sickness risk factors, including excessive blood pressure, a record of coronary heart disorder within the family, pressure, age, sex, levels of cholesterol, body mass index (BMI) and poor dwelling choices. The hybrid technique makes use of the nice functions of LR for clean understanding, SVM for dealing with large amounts of facts and NNs for finding developments. By integrating these models together, the HMLCRP makes positive that type is correct and that danger predictions are accurate. In this study, benchmark datasets used, which include the cardio statistics set, heart ailment dataset and Framingham heart examination dataset, are used to train and test the version. Popular parameter measures, such as accuracy, precision, recall and the F1‐score, are used to determine overall performance. The results of the experiments indicate that the HMLCRP is better at predicting effects than individual models. The suggested combination model is a major step forward in personalised healthcare because it allows proactive risk management and early intervention methods to stop CVD.

## 1 Introduction

The main cause of death worldwide is cardiovascular diseases (CVDa), which affects approximately 18 million people a year and accounts for approximately 31% of all deaths worldwide. Heart disease, stroke and heart failure are just a few of the diseases that are caused mostly by a mix of genetic, behavioural and environmental factors. High blood pressure, high cholesterol, being overweight, smoking, worrying, not being active and having a family background are all major risk factors. CVDs are becoming more common, and healthcare systems have to address more patients. This shows how important it is to have advanced risk prediction gear, which can assist in locating issues early and preventing them before they worsen. Although scientific scoring structures and statistical models are often used in conventional ways for identifying cardiovascular risk, they are not very proper at displaying how specific factors interact with each other. Machine learning (ML) is a game‐changing option that makes use of data to make predictions more accurate, sort patients into groups and help with personalised treatment plans. ML algorithms have been very beneficial in scientific prognosis, including the use of large amounts of patient data to locate trends and make very accurate predictions about how diseases will develop. However, there is not always a single ML model that can appropriately measure all the elements associated with the likelihood of CVD. We suggest a hybrid ML technique for cardiovascular risk prediction (HMLCRP) to address this risk. This suggested method combines several ML models, including logistic regression (LR), support vector machines (SVMs) and neural networks (NNs). Each of these models has its own strengths that help them make better predictions. LR is a type of class that is typically used in medicine. It can be used to determine how likely it is that a person may have a circulatory ailment. It is easy to understand, so medical doctors can determine how positive risk factors affect patients. SVMs are good at working with large quantities of data that cannot be separated in a direct line. This makes them useful for analysing complicated heart records sets. NNs are important for determining complicated links between elements. They are able to make greater accurate predictions by means of learning deep feature representations from data. The suggested hybrid method takes the quality parts of both of these models and makes use of them together to make cardiovascular risk assessment better. First, the data are preprocessed to ensure that important risk elements such as sex, age, blood pressure, cholesterol, BMI, family history, stress levels and lifestyle choices are taken into consideration. Next, each model (LR, SVM and NN) is trained independently so that the overall performance of each may be analysed. The hybridisation process takes the results of both the LR and the SVM and feeds them into the NN as features. This allows the NN to make better predictions based on the basis of both linear and nonlinear relationships. This organisational approach improves the overall accuracy of predictions, making the model for assessing cardiovascular risk stronger and more accurate.

The proposed technique is tested on benchmark datasets including the cardio dataset, heart disease database and Framingham heart study dataset. There are many medical records in these databases including identified cardiovascular risk factors. This allows us to test the mixed model in many various ways and train it via many approaches. Standard classification measurements, such as accuracy, precision, recall and F1‐score, are used to measure performance. The results of the experiments show that the HMLCRP does better than individual ML models by being more accurate and stable in its predictions. This makes it a potentially useful tool for identifying cardiovascular risk early in life. This study offers a good way to perform personalised risk assessment and proactive disease control by combining several ML methods. Healthcare workers might be able to use the suggested combination model to help with early identification, focused treatments and better patient results. In the future, more work will be done to improve the prediction accuracy and practical usefulness by adding deep learning methods and increasing the dataset size.

The contribution of this article is as follows:

- This paper proposes the HMLCRP, a hybrid model that combines LR, SVMs and NNs for increased CVD risk prediction.
- It incorporates key risk factors such as high blood pressure, family history, stress, age, sex, cholesterol, BMI and unhealthy lifestyle choices for more accurate predictions.
- The model is validated via benchmark datasets, including the cardio dataset, heart disease dataset and Framingham heart study dataset, ensuring reliability.
- The experimental results show higher accuracy, precision, recall and F1‐score than those of individual models do, improving CVD classification.
- The study contributes to personalised healthcare by enabling early diagnosis and proactive interventions, with future improvements through deep learning integration and dataset expansion.

The remainder of this paper is structured as a literature review present in Section 2 which presents system learning models for CVD prediction, hybrid methods and study gaps. Section 3 describes the proposed method and Section 4 describes the proposed approach technique, which includes facts preprocessing, ML models (LR, SVM, NN) and the HMLCRP framework. Section 5 presents the experimental results, function importance, medical relevance and demanding situations. Section 6 concludes with key findings for enhancing CVD risk prediction.

## 2 Related Work

Heart and blood vessel diseases are among the main causes of death worldwide. Therefore, we need accurate and useful prediction models to help with early identification and risk assessment. Researchers are looking into different computer methods for identifying CVD risk because more healthcare data are becoming available and ML is improving. However, it can be difficult for current models to address large datasets, select important traits and make predictions more accurate. ML and deep learning methods such as ensemble models, feature selection approaches, deep NNs and optimisation strategies have been used in various papers to solve these problems. This discusses previous work that has been done in this area, with a focus on current ML models, hybrid methods and optimisation techniques. It also discusses approximately the gaps that the recommended hybrid ML technique for cardiovascular risk prediction (HMLCRP) desires to fill. Several studies have checked out the way to use ensemble and hybrid models to get better at predicting cardiovascular risk. In 2024, Vijayaraj and Pasupathi [1] created the Hybrid Harris hawks optimisation (H‐HHO) approach, which mixes several devices studying models, consisting of SVMs, LR and random forest (RF). The better accuracy they achieved with their technique was 94.74%, showing that mixed models can improve classification capacity. In the same way, Pasha and Mohamed [2] proposed a novel function reduction (NFR) model that uses ML and data mining together to make disorder risk forecasting better. Their technique focused on reducing the number of dimensions of the features while maintaining the accuracy high (95.52%) and a high area under the curve (AUC) (99.20%), showing how essential feature selection is in predictive modelling.

A number of people have extensively utilised deep learning to anticipate CVD. These strategies use neural network designs to find complicated developments in scientific statistics. An optimised scientific characteristic analysis framework used to be created via Alwakid et al. [3]. This framework used deep learning‐based feature selection to cut down on human entry and improve model performance. Their method had an AUROC of 91.30%, which makes it a beneficial device for making remedy selections. In the same method, Vyshnya et al. [4] integrated convolutional neural networks (CNNs) with internet of clinical matter (IoMT) structures so that clinicians may want to become aware of coronary heart ailment in real time. This examination focused on how deep learning might be used to improve customised healthcare. Several papers have mentioned how essential feature selection strategies are for making models work better. Asha and Ramya [5] used the artificial flora algorithm with a SVM to sort CVDs, showing how nature‐inspired optimisation can help choose higher functions and increase the accuracy of classifiers. In the same method, Ghosh et al. [6] used the relief and LASSO feature selection methods, which showed that lowering functions effectively can greatly increase version overall performance and readability. Heart sickness detection has proven promise with hybrid ML models that integrate a couple of predictors. In study by Khan et al. [7] created a blending‐based ensemble model to find out if patients are secretly smokers, which is a key thing in their risk of coronary heart ailment. Their technique showed that the usage of ensemble gear may want to assist figure out which patients are at the highest risk. In addition, Abrar et al. [8] advised a multi‐agent technique for personalised hypertension threat prediction that uses affected person data and ML methods to increase the accuracy of predictions. These studies display that using more than one model together can assist increase the effects of CVD type. Several research have also appeared into computerised healthcare decision assist systems which can be based totally on deep gaining knowledge of. A have a look at by way of Alshwaheen et al. [9] suggested using LSTM‐RNN to are expecting when an affected person in the intensive care unit (ICU) will get worse. This indicates that deep learning is better at predicting the future than traditional device studying models. Similarly, Chushig‐Muzo et al. [10] created a GAN‐based model to are expecting cardiovascular chance, the use of strategies for creating faux statistics to make the model more reliable. The usage of advanced deep learning models collectively has proven lots of promise in improving cardiovascular healthcare via making early identification and risk evaluation better.

Many studies have long focused on improving ML‐based CVD forecast models better via optimisation methods. Rahim et al. [11] created a mixed Harris Hawks optimisation model for predicting coronary artery ailment that was once more accurate than other optimisation methods at classifying patients. In the same way, Lai et al. [12] used context‐conscious optimisation techniques, such as Harris Hawks and swarm intelligence algorithms, to increase models that predict CVD. These optimisation strategies have been shown to substantially improve the efficiency of models and the accuracy of predictions. These days, researchers have also been searching into how to use explainable AI (XAI) methods in models that are predicting coronary heart disorder. Ghorashi et al. [13] used SHAP‐based feature selection and AI tools that could be explained to make models clearer and build faith within the scientific world. Their study showed how essential it is for ML models to be easy to understand so they can be utilised in real‐life healthcare situations. Also, Sunilkumar and Kumaresan [14] created an explainable decision support system that gave every patient a customised risk assessment, which made AI‐based healthcare solutions simpler to use. Currently, researchers have also investigated risk prediction models for CVD screening that are driven by ML. Kuang et al. [15] created a CVD forecast system using an adaptive genetic algorithm and fuzzy logic. This showed that combined computational intelligence strategies could substantially enhance the accuracy of risk classification. As an example, Monda et al. [16] created a mixed prediction model for clinical datasets by optimising model generalisability via making the best use of data splitting and feature tuning. Their work suggests how important combined strategies are for solving problems such as uneven data and redundant features. Some research projects have looked into how to use mobile and IoT to make CVD risk prediction models that can be used for real‐time tracking and early intervention. Nancy et al. [17] proposed an IoT‐based total risk assessment model that could use sensors to accumulate data and cloud‐based analytics to monitor human heart health all of the time. Their approach showed that real‐time ML models could make detecting risks early on and take preventative healthcare actions. Despite these enhancements, current ML models for predicting CVD still have difficulty selecting the right features, are easy to understand and are capable of being used in other situations. Deep learning‐based models are more accurate, but they are not always clear, which makes it difficult for clinicians to use them. Conventional ML models, however, such as LR and SVM, are easier to understand, but they have trouble with complex, high‐dimensional data. To address these problems, hybrid strategies have been counselled that blend the best parts of several ML strategies. In Table 1, studies on CVD prediction models are compared, with data about datasets, feature counts, methods, accuracy and bounds shown. With accuracy rates between 88.9% and 97.2%, the research uses ML, deep learning and optimisation techniques. However, there are a few issues with how easy they are to recognise, how much computing power they need, how challenging it is to select the right features and the size of the datasets they use.

**TABLE 1:** Comparison of previous studies and their limitations.

Author, year	Dataset targeted	No. of features used	Algorithm	Accuracy (%)	Limitation
Vijayaraj and Pasupathi, 2024 [1]	Heart disease dataset	15	Harris Hawks + SVM	94.74	Limited interpretability
Pasha and Mohamed, 2020 [2]	Clinical feature dataset	12	Feature reduction + ML	95.52	Feature reduction may discard vital data
Alwakid et al., 2025 [3]	Optimised clinical data	10	Deep learning + feature selection	91.3	Limited dataset scope
Vyshnya et al., 2024 [4]	IoMT medical data	20	CNN on IoMT	89.8	Requires high computational power
Asha and Ramya, 2025 [5]	CVD classification data	14	Artificial flora + SVM	92.45	Optimization complexity
Ghosh et al., 2021 [6]	Relief and LASSO feature data	11	Relief + LASSO ML	90.12	Feature selection constraints
Khan et al., 2024 [7]	Smoking and CVD data	18	Blending ensemble ML	93.25	Complex ensemble structure
Abrar et al., 2021 [8]	Hypertension risk data	16	Multi‐agent ML	94.5	Multi‐agent approach needs refinement
Alshwaheen et al., 2021 [9]	ICU patient data	9	LSTM‐RNN	96.3	Limited to ICU settings
Chushig‐Muzo et al., 2024 [10]	Diabetic CVD dataset	13	GAN‐based model	91.75	GAN data may lack real‐world validity
Rahim et al., 2021 [11]	Coronary artery disease	12	Hybrid Harris Hawks	94.8	Not tested on large populations
Lai et al., 2019 [12]	Sudden cardiac death data	15	Context‐aware optimization	92.5	Context dependency affects results
Ghorashi et al., 2023 [13]	CVD symptom dataset	14	Regression + GAN Model	90.3	Regression may not capture complex patterns
Sunilkumar et al., 2024 [14]	Cardiology deep learning data	10	Transfer learning + deep learning	88.9	Deep learning models lack interpretability
Ganapathy et al., 2022 [18]	CNN, RNN, GAN dataset	20	CNN, RNN, GAN hybrid	92.95	CNN/RNN/GAN hybrid lacks real‐world testing
Cai et al., 2024 [19]	AI‐based risk assessment data	21	AI predictive model	94.5	AI model needs clinical validation
Sathasivam et al., 2025 [20]	Harris Hawks optimised data	18	Harris Hawks‐based optimization	93.8	Optimization approach needs larger dataset

The proposed hybrid system studying technique for cardiovascular threat prediction (HMLCRP) builds on previous paintings with the aid of combining LR, SVMs and NNs. This makes the predictions more correct, easier to recognise and beneficial in clinical settings. The model makes use of the great functions of LR to make it smooth to understand, SVM to address massive amounts of records and NNs to discover complicated, nonlinear connections. With combining those models, the HMLCRP makes positive that classification is accurate and that threat estimates are correct. Benchmark datasets such as the cardio statistics set, heart sickness dataset and Framingham heart observe dataset are used to educate and check the model. This makes positive that it can be used in real‐existence clinical conditions. Well‐known type variables such as accuracy, precision, recall, F1‐rating and AUROC are used to measure overall performance, displaying that it is higher than character ML models.

### 2.1 Gap in Research

CVD is still one of the principal causes of death worldwide. To help with early identification and risk evaluation, we need to create accurate and beneficial prediction models. ML strategies have made large steps ahead in predicting CVD. However, contemporary models nonetheless have primary flaws that make them difficult to apply in real‐life medical settings. One of the principal issues is that they are no longer very generalisable; selecting functions is difficult, it is challenging to apprehend and it is difficult to compute. These problems indicate that we want a broader combined approach that combines the fantastic parts of various systems to gain knowledge of models to make predictions more correct, dependable and smooth. Too much dependence on standby ML models, which regularly fail to disclose how complex CVD risk factors are connected, is one of the largest gaps in modern research. As an example, popular models such as LR are easy to understand; however, they can notfind connections within the data that are not linear. Support vector machines (SVMs), then again, work properly in high‐dimensional spaces but not so properly with large datasets because they are very costly to run. Neural networks (NNs) also are very accurate at finding complicated patterns and deep feature representations; however, they regularly behave like “black container” models, which make them difficult to apply in scientific settings because they are not clear. This approach can use the interpretability of LR, the stability of SVMs in high‐dimensional data and the deep pattern recognition power of NNs. Choosing features and lowering the number of dimensions is another area that needs more study. Many of the current CVD forecast models use a lot of input data, which can make them more expensive to run and make them more likely to be too good at what they do. Feature selection methods, such as Relief and LASSO‐based methods, are easier to understand, but leaving out some factors could mean that important forecast information is lost. Optimisation algorithms that are based on nature, such as Harris Hawks Optimisation (HHO) and Artificial Flora Algorithms, have shown promise in making feature selection more efficient, but they haven't been widely used in mixed ML systems yet. A complete mixed method should include smart feature selection tools that make models more efficient without lowering their accuracy.

To make predictions more accurate, ensemble learning and multi‐agent systems have also been looked into. In cardiovascular danger class, mixing‐based ensemble models and multi‐agent systems for high blood pressure risk prediction. However, these methods are not very flexible in terms of different datasets, and they regularly need quite a lot of hyperparameter tuning, which makes them less useful for real‐time clinical utilisation. To make things more flexible and scalable, we need a blended ensemble ML technique that chooses and improves models based on real‐world clinical data. Deep learning‐based CVD prediction has also been regarded with the usage of long short‐term memory (LSTM) networks to predict how a patient will worsen and generative adversarial networks (GANs) to add new records. The accuracy of these models could be very good; however, they need a variety of labelled records, which are not available in clinical settings. In addition, deep learning models are difficult to apply in healthcare settings with limited resources because they are computationally costly and require many tools. To make deep learning models more beneficial in the real world, future studies need to focus on mixed deep learning systems that combine feature selection, ensemble ML and interpretable AI strategies. Another important issue is the lack of customised models that can predict CVD risk in real time. A variety of studies have examined historical records with a preference for keeping an eye on patients in real time. The use of IoT and AI‐driven models together has made it possible to monitor health in real time, but there are still issues with safety and data privacy. A comprehensive blended model should encompass IoT‐enabled AI frameworks with robust security and secure data‐sharing selections to make assessing cardiovascular risk in real time easier while maintaining patient privacy. Utilising data systems for real‐time patient monitoring and diagnosis emphasises the enhancement of healthcare efficiency and patient outcomes through intelligent management systems. Explainability and clinical interpretability are still large issues that make it difficult for ML models to be broadly utilised in healthcare. Many doctors are hesitant to trust ML predictions as it is challenging to see how decisions are made in complex AI‐driven models. Explainable AI (XAI) techniques, such as SHAP‐based feature selection and interpretable deep learning have made some models clearer, but more work needs to be done to create hybrid models that can explain clinical decisions in real time and for every patient.

## 3 Proposed Method: Hybrid Machine Learning Approach for Cardiovascular Risk Prediction (HMLCRD)

The proposed method suggests a hybrid ML approach for cardiovascular risk prediction (HMLCRP) to address problems identified in earlier research studies. This technique improves the accuracy, readability and stability of CVD risk prediction [21]. The advised approach focuses on preparing the facts, deciding on the right functions, building a mixed model and carefully checking its overall performance with the purpose of offering a whole and strong framework for making predictions [22]. An essential feature of scientific datasets is that they frequently lack statistics or are inconsistent due to variations in medical information. To address this issue, we use legitimate imply replacement and outlier evaluation methods to fill in missing numbers Dodeen [23]. Normalisation methods such as min‐max scaling and standardisation are also used to ensure that everyone affected person facts are the identical [24]. Selecting the proper functions is a key part of reducing the number of dimensions and making the model work efficiently. A few antique oversampling strategies, such as SMOTE and canopy‐based k‐capacity clustering [25], have been used to cope with elegance mismatches. However, those techniques can sometimes add noise, which could lead to class errors. This is why our model uses a mixed feature selection method that blends the Relief and LASSO approaches to keep only the most useful features.

Our suggested model aims to improve the ability to identify the risk of CVD by including patient characteristics that are practically important. Blood pressure is one of the main causes, since high blood pressure is known to lead to heart problems. People with a family history of CVD are more likely to get it themselves. This is because of genetic predisposition, which makes it an important factor for accurately estimating risk. Worry levels are also very important for heart health because long‐term worry can lead to high blood pressure and other metabolism problems. Age and gender are also important biological factors because getting older naturally makes you more likely to get CVD, and differences between men and women affect risk stratification. Cholesterol values, especially low‐density lipoprotein (LDL) and high‐density lipoprotein (HDL), are also very important because they are key signs of heart disease. Body mass index (BMI) is looked at because metabolic syndrome and fat are directly linked to heart problems. Finally, bad habits such as food choices, smoking and exercise are included in the model because they greatly raise the chance of heart disease. By using these different but connected factors, our method makes sure that the risk of CVD is assessed in a way that is both personalised and based on data. This leads to better ways to avoid CVD and early action. Our model uses a mixed ML method, which means it combines several models that use different computer skills to make more accurate predictions [26]. LR is included because it is commonly used in medical calculations, as it can estimate probabilities and be easy to understand. LR works best for cases with two options, such as telling the difference between people with high risk and those with low risk. Support vector machines (SVM), on the other hand, work well with high‐dimensional clinical records and handle complex, nonlinearly separable patterns well. This is because LR has trouble with high‐dimensional datasets. Neural networks (NNs), especially feedforward neural networks, are used to improve prediction even more. Not only can NNs capture complex, nonlinear relationships in the dataset, but they are also good at finding complicated circulatory trends that other models might miss. Putting LR, SVM and NN together makes a strong mixed prediction model that strikes a good balance between being easy to understand, being scalable and being able to find deep patterns in CVD risk assessment.

**FIGURE 1:**
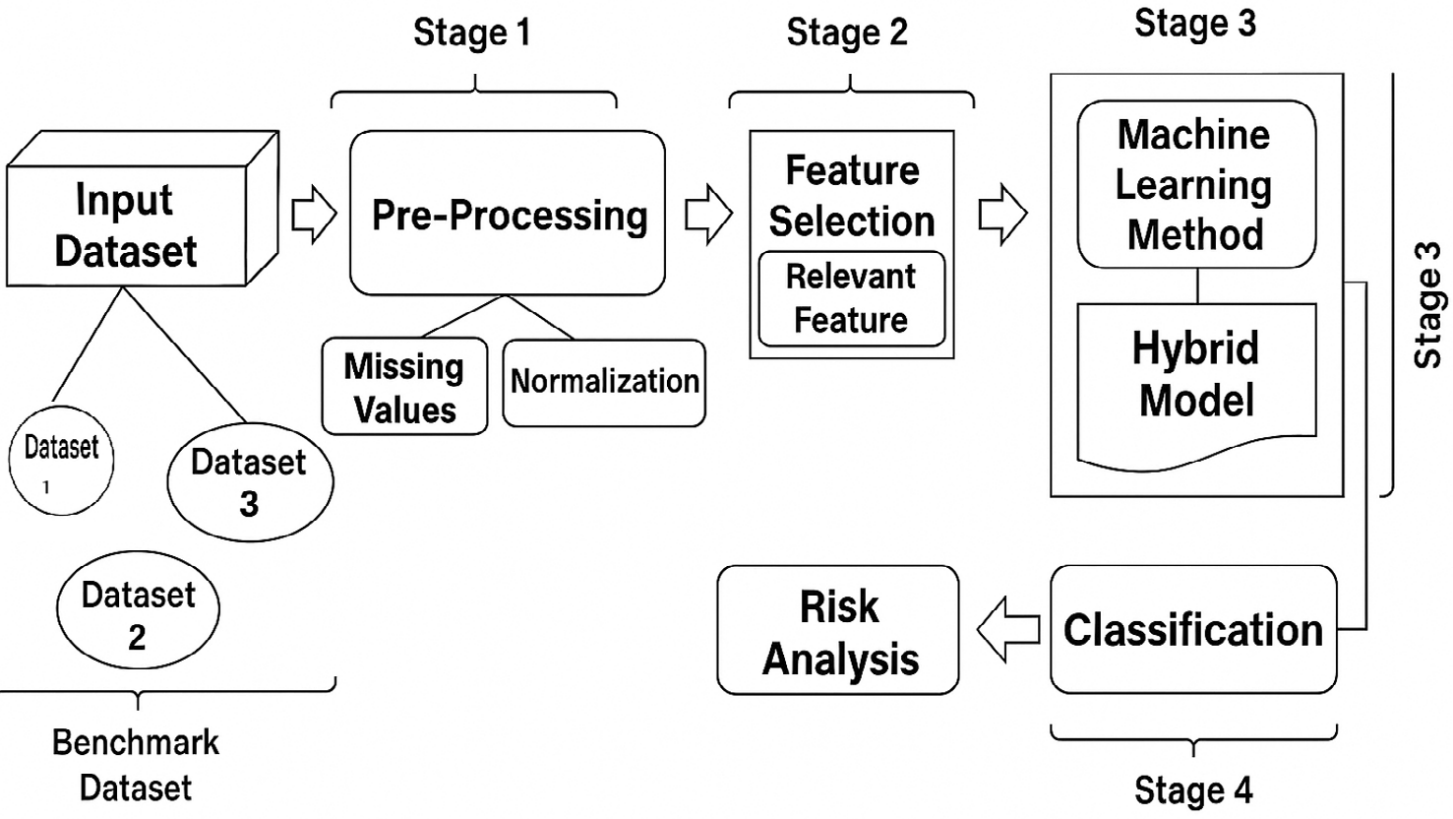
Representation of the proposed CVD risk analysis framework for the HMLCRP.

The hybrid ML approach for cardiovascular risk prediction HMLCRP uses a technique called “ensemble learning” to improve model performance. The suggested hybrid ML approach for predicting CVD risk is shown in Figure 1. It has four steps: (1) preprocessing, which includes dealing with missing values and normalising the data; (2) feature selection to find important traits; (3) ML and hybrid model development to make final predictions; and (4) classification and risk analysis to see how accurate the predictions are. First, the results of LR and SVM are calculated and used to explain functions in a central degree. The neural network (NN) then receives these outcomes as more statistics. This lets the NN make higher guesses model learning of each linear and nonlinear connection within the records. The relief and LASSO function selection techniques are used to eliminate pointless characteristics and accelerate computations for feature extraction and weighted combination. It calculates a weighted blend of LR, SVM and NN effects to ensure that each model's expected input is at its best based totally on how properly it skilled. This combination makes up for the failings of each version by way of mixing LR's ease of use, SVM's reliability in large datasets and NN's complicated pattern recognition competencies. As a result, HMLCRP is extremely accurate and may be used in extra conditions, so it could be used in therapeutic settings. We perform many checks with common classification measures to make sure that the HMLCRP works. Accuracy measures how properly estimates are made basic, whilst accuracy and memory measure how well the model reveals actual positives. The F1‐score gives an equal weight to accuracy and memory, making sure that the version does not favour one over the other. It's also possible to use ROC‐AUC to see how properly the model can inform the difference between people who have CVD and those who do not. This gives us and thinks of how well it could discriminate.

## 4 Methodology

### 4.1 Dataset Used

#### 4.1.1 Dataset 1: Cardio Dataset

There are 70,000 patient records from medical exams in the cardio Dataset, which is a popular collection for predicting CVD [27]. There are 11 important clinical factors, such as age, sex, height, weight, systolic blood pressure, diastolic blood pressure, cholesterol levels, glucose levels, smoking status, alcohol intake and level of physical exercise. The dataset has a binary classification labelled with 0 for no CVD and 1 for the presence of CVD. This makes it good for guided ML tasks. The data comes from regular medical check‐ups, so risk forecast tools can be used in the real world. One problem with this information is that it is not balanced between classes because many of the records are from people who do not have CVD. To improve model performance, we need to use preparation methods such as SMOTE (synthetic minority oversampling technique) or class balancing. Feature selection methods such as Relief and LASSO can be used to find the best models for determining cardiovascular risk.

#### 4.1.2 Dataset 2: Heart Disease Dataset (Cleveland)

The Heart Disease Dataset [28] comes from the UCI Machine Learning Repository and has more than 900 records from different sets, such as the Cleveland, Hungarian and Switzerland datasets. In this study, we considered only the Cleveland dataset. It has 14 main parts, such as age, gender, resting blood pressure, cholesterol levels, fasting blood sugar, maximum heart rate, exercise‐induced angina, ST depression, major vessel count and resting electrocardiographic results. The information is labelled with numbers from 0 to 4, where 0 means there is no disease and 1 to 4 means that the severity of heart disease is increasing. Data enrichment and feature engineering methods are often used to improve forecast performance because the dataset is not very large. The dataset has been widely used in ML research because it is well‐structured and has a good mix of clinical and population risk factors for predicting CVD.

#### 4.1.3 Dataset 3: Framingham Heart Study Dataset

The Framingham Heart Study Dataset [29] is one of the most complete sets of data for predicting CVD risk because it is an ongoing group study with data gathered over many decades. Fifteen important risk factors in its 4240 records, such as age, sex, smoking habits, diabetes, systolic and diastolic blood pressure, cholesterol levels, BMI, heart rate and glucose levels were identified in the 4240 records. The collection is very useful because it includes both short‐term and long‐term cardiovascular results. This allows researchers to look at how CVD gets worse over time. One problem with this dataset is that it has some lost data because of long‐term follow‐ups, which means that good interpolation methods are needed. It also has factors that change over time, which means that more complex models such as long short‐term memory (LSTM) networks or time‐series analysis methods are needed. The dataset is perfect for making predictive models for the early diagnosis of CVD and preventive measures because it has a lot of historical records. The Framingham dataset has been very helpful in creating standard risk score models, such as the Framingham risk score, which is still used as a standard for determining cardiovascular risk [30].

### 4.2 Data Collection and Preprocessing

This is an important set up as a strong data collection and preprocessing pipeline to ensure that our hybrid ML approach for cardiovascular Risk Prediction (HMLCRP) works well and accurately. The cardio dataset, heart disease dataset and Framingham heart study dataset are three well‐known standard datasets that are used in this study [31]. These sets of data include a wide range of patient records that include personal details, clinical factors and living habits. All of these are important for correctly estimating cardiovascular risk. However, raw healthcare data often have missing values, errors and different scales, so they need to be processed in a planned way to make the model work better and be more reliable. We used various estimation methods to address the problem of missing numbers. To keep the data consistent, mean and median imputation were used for numeric characteristics [32]. K‐nearest neighbours (KNN) imputation, on the other hand, filled in null values based on how similar the patient profiles were. Also, regression‐based estimation was used to guess missing values by using features that are linked to each other, keeping the links between features intact. Outlier identification methods, such as *Z*‐score analysis and interquartile range (IQR) filtering, were used to ensure the quality of the dataset. These methods found and removed high values that could negatively affect the model's ability to predict the future.

**FIGURE 2:**
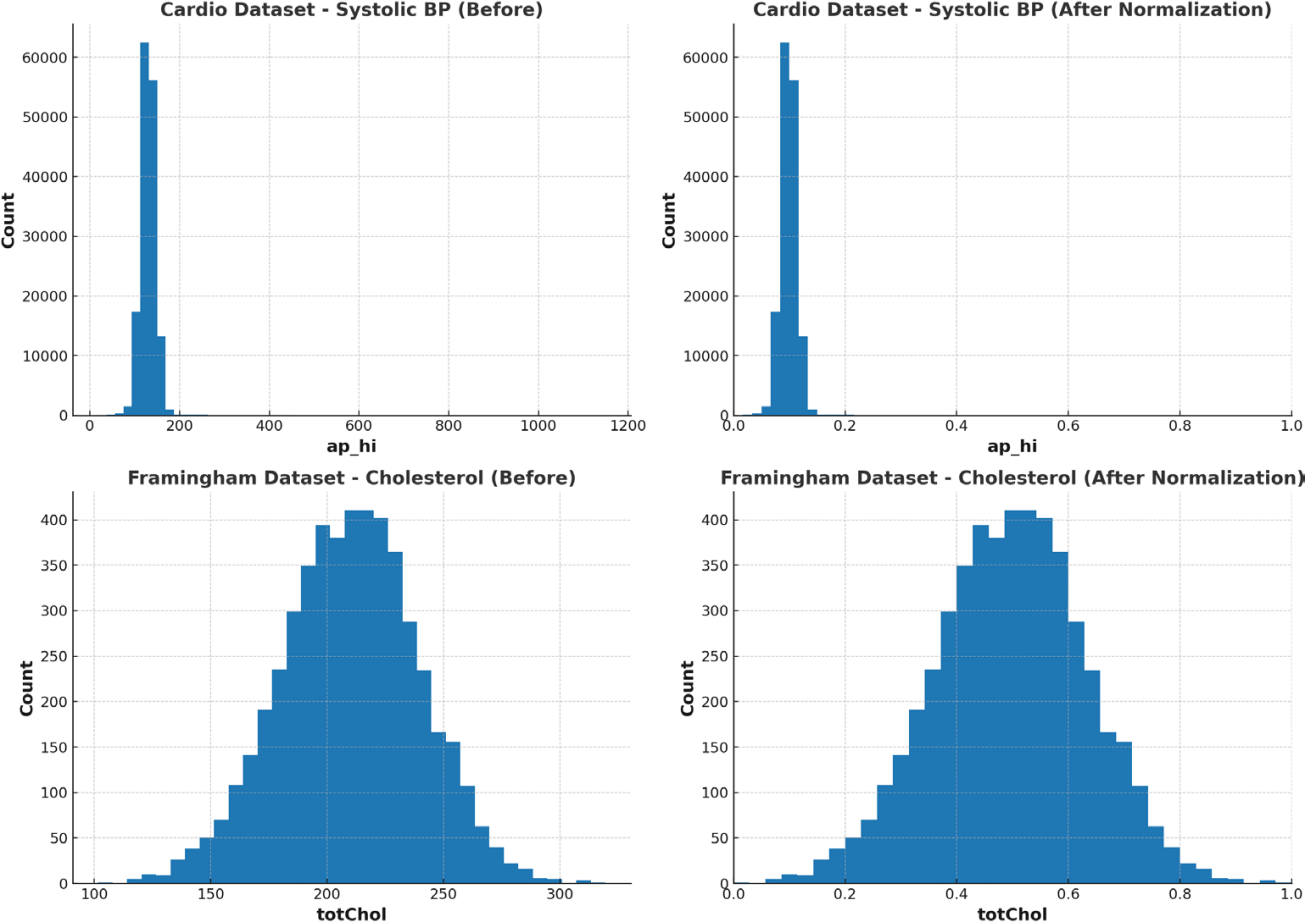
Cholesterol distribution dataset 1 and dataset 3 (after normalisation).

Normalisation methods, such as min‐max scaling and *Z*‐score normalisation, were used to ensure that all of the traits were the same (Figure 2 shows the after‐normalisation effect). Because clinical measurements differ in sizes, these methods prevent traits with higher values from having an unfair effect on the learning process. Additionally, feature selection was carried out to improve model generalisability and make computations more efficient [33]. We used Relief and LASSO (Least Absolute Shrinkage and Selection Operator) to eliminate traits that were already present while keeping the most important ones that predicted cardiovascular risk. We made sure that the ML model was trained on the most important characteristics by improving the dataset. This improved both accuracy and readability [34].

**FIGURE 3:**
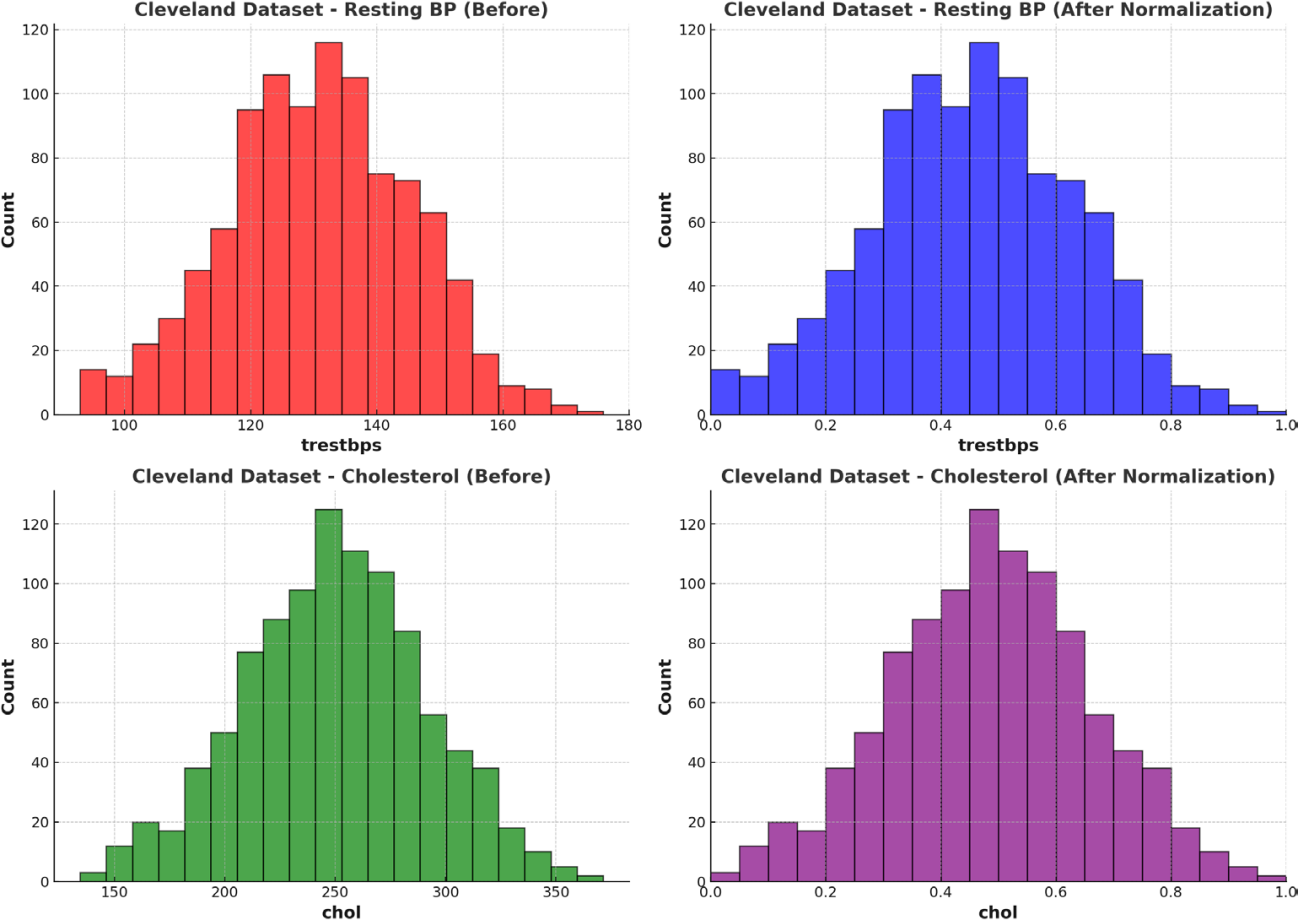
Cleveland dataset—cholesterol (after normalisation).

The final set of data we used included highly important risk factors that have been proven repeatedly in cardiovascular studies. Some of these are high blood pressure (hypertension), which is still one of the main reasons for heart disease and family history, which indicates that some individuals are genetically more likely to get heart disease. Stress levels were looked at because they are linked to metabolic diseases and high blood pressure. Men and older people are usually more likely to have heart disease, so age and gender were included because they play a big role in that risk. LDL and HDL cholesterol values were used as main indicators for heart disease, and body mass index (BMI) was used to measure fat and metabolic health, after preprocessing analysis for dataset 2 shown in Figure 3. We also looked at living habits, such as smoking, drinking alcohol, eating habits and physical exercise, because these all have a big effect on cardiovascular health. We made sure that our dataset is organised, of high quality and best for ML methods by using data cleaning, normalisation and feature selection techniques successfully. The preprocessed data constitute a solid basis for the HMLCRP method, which greatly improves the accuracy of predictions and makes it easier to create a useful model for examining the risk of CVD and developing early intervention strategies.

### 4.3 Hybrid Feature Selection Relief and LASSO

Combining hybrid relief and LASSO is a way to select features that make ML models more accurate and efficient. Relief is a filtering‐based approach for identifying how important a characteristic is by guessing how well it tells the distinction between close by times of different classes. It helps discover the important qualities while ignoring the vain or demanding equalities. The Least Absolute Shrinkage and Selection Operator (LASSO) is a regression‐based method that makes use of L1 regularisation to make some values zero. While hybrid relief and LASSO are used collectively, they effectively lower the number of dimensions while preserving important records. This approach makes models easier to recognise, quickens computations and guarantees greater effects in predicting cardiovascular threat.

**Algorithm for Hybrid Relief and LASSO Feature Selection**

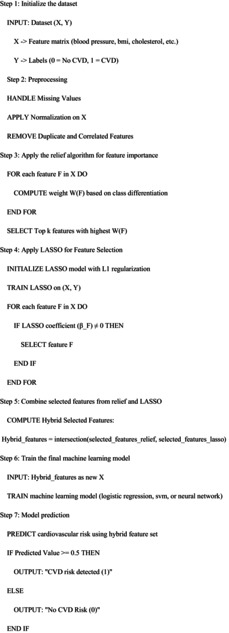

### 4.4 Machine Learning Models

#### 4.4.1 Logistic Regression

LR is a popular supervised learning method for binary classification tasks. This makes it a great choice for predicting the risk of CVD. One of the best things about it is that it is easy to understand because it gives doctors a simple probability result that helps them see how separate variables (risk factors) affect the chance of CVD occurring. LR models the log‐odds of the chance of an event occurring as a linear mix of input traits. This makes it a useful tool for determining risk on the basis of factors such as blood pressure, cholesterol levels, body mass index (BMI) and lifestyle choices. In this research, LR was added to the hybrid ML approach for cardiovascular risk prediction (HMLCRP) to make it easier to understand and to help identify more complicated models. LR is very good at finding linear connections in the data, which makes it easy to see how each risk factor affects the chance of developing CVD. However, it does not work as well when dealing with high‐dimensional, nonlinear and complex relationships between traits, though. In our suggested hybrid model, we address this problem by mixing LR with support vector machines (SVM) and neural networks (NNs). Regularisation methods such as L1 (LASSO) and L2 (ridge regression) were also used to stop overfitting and ensure the results were accurate. Even though it is simple, LR is still an important part of our model because it is easy to use, does not cost much to run and can provide clinically useful risk estimates for predicting CVD.

**Algorithm: Logistic Regression for Cardiovascular Risk Prediction**

Step 1: Compute the sigmoid function
$$h_{\text{theta}\left(x\right)}=\frac{1}{1+e^{-{\text{theta}}^{T}*x}}$$
  Converts linear output into probability between 0 and 1, determining such likelihood of cardiovascular disease presence.
Step 2: Define the hypothesis function
PY=1X=1/1+e^−theta_0+theta_1*x_1+…+theta_n*x_n
  Predicts probability of cardiovascular disease based on patient features such as age, BMI and cholesterol levels.
Step 3: Compute the cost function (log loss)
$$J\left(\text{theta}\right)=-\left(\frac{1}{m}\right)*\mathrm{sum}\left(\;y_{i}*\log\left(h_{\text{theta}\left(x_{i}\right)}\right)+\left(1-y_{i}\right)*\log\left(1-h_{\text{theta}\left(x_{i}\right)}\right)\right)$$
  The model's prediction error is measured, penalising incorrect classifications and ensuring better cardiovascular risk prediction.
Step 4: Apply the gradient descent for parameter optimisation
$$\text{theta}\_j≔\text{theta}\_j-\text{alpha}*\left(1/m\right)*\mathrm{sum}\left(\;\left(h\_\text{theta}\left(x\_i\right)-y\_i\right)*x\_\mathrm{ij}\;\right)$$
  The weights are updated iteratively to minimise cost, improving the prediction accuracy in cardiovascular disease classification.
Step 5: Establishing the decision boundary
$$\begin{array}{c} y\_\mathrm{hat}=\{\;1,\text{if}\;h\_\text{theta}\left(x\right)\geq 0.5\\ 0,\text{otherwise}\;\} \end{array}$$
  Classifies patients as high‐risk (*y* = 1) or low‐risk (*y* = 0) based on computed probability.

#### 4.4.2 Support Vector Machines (SVM)

Support vector machines (SVM) are strong ML models that work well with large datasets. This makes them an outstanding choice for identifying the chance of CVD. SVM works by means of finding a satisfactory hyperplane that divides classes. This is extraordinary from LR, which assumes that input tendencies and outputs are related in a direct line. This is especially beneficial when working with records that cannot be separated in an immediate line, such as while there are many complicated connections between coronary heart disease risk elements. SVMs make use of kernel functions, such as the radial basis function (RBF), to transport uncover records into places with more dimensions in which a linear separator may be located. This makes it less difficult to classify things. We recommend the use of a hybrid ML method for cardiovascular risk prediction (HMLCRP). SVM is a key part of this method because it enables us to address complex, high‐dimensional scientific records. Many elements, such as age, levels of cholesterol, body mass index (BMI) and stress levels, are connected in the manner of predicting CVD. SVM helps to improve classification by reducing classification errors and increasing the distinction between hazard groups. One high‐quality component about SVM is that it does not get too good at fitting, even if there are too many functions compared to samples. But SVM models may be challenging to compute and need hyperparameter tuning (as an example, kernel selection and regularisation parameter C) to work at their best. We used grid search and cross‐validation to quality‐track the SVM settings. By combining SVM with LR and neural networks, we have been capable of using its high‐dimensional classification capabilities even while keeping clarity and processing speed in mind. This makes it an essential part of our strategy for predicting CVD risk.

**Algorithm**

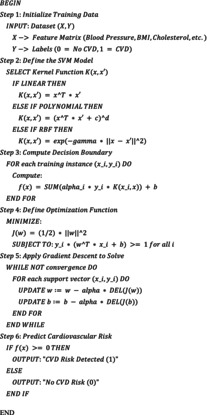

#### 4.4.3 Neural Networks (NNs)

Neural networks (NNs) are deep learning models that could locate complex, nonlinear connections in records. This makes them very beneficial for predicting the risk of CVD. NNs use a hierarchical community of neurons to study patterns immediately from statistics, whilst LR and support vector machines use mathematical features that have already been installed. This feature allows NNs to perform complicated connections among scientific and normal risk elements, which substantially improves the accuracy of predictions. NNs are essential in our hybrid ML approach for cardiovascular risk prediction (HMLCRP) because they assist us summarise functions and discover deep patterns. Our neural community version makes use of many hidden layers to extract important styles from very massive clinical data units. This shallows it to locate small developments that different models might leave out. Activation features such as sigmoid and ReLU (rectified linear unit) have been used to introduce nonlinearity, and batch normalisation and dropout regularisation have been used to prevent the model from fitting too well. One of the principal issues with NNs is that they are tough to recognise and challenging to apply as compared to less difficult models such as LR. However, our combination approach, which combines SVM and LR, provides a great blend between accuracy, clarity and computing pace. We ensure that minor cardiovascular risk trends are captured through the inclusion of NNs in the prediction model. This advanced the overall accuracy and memory of our framework for early CVD prognosis and hazard stratification.

### 4.5 Hybrid Model Development (HMLCRD)

**FIGURE 4:**
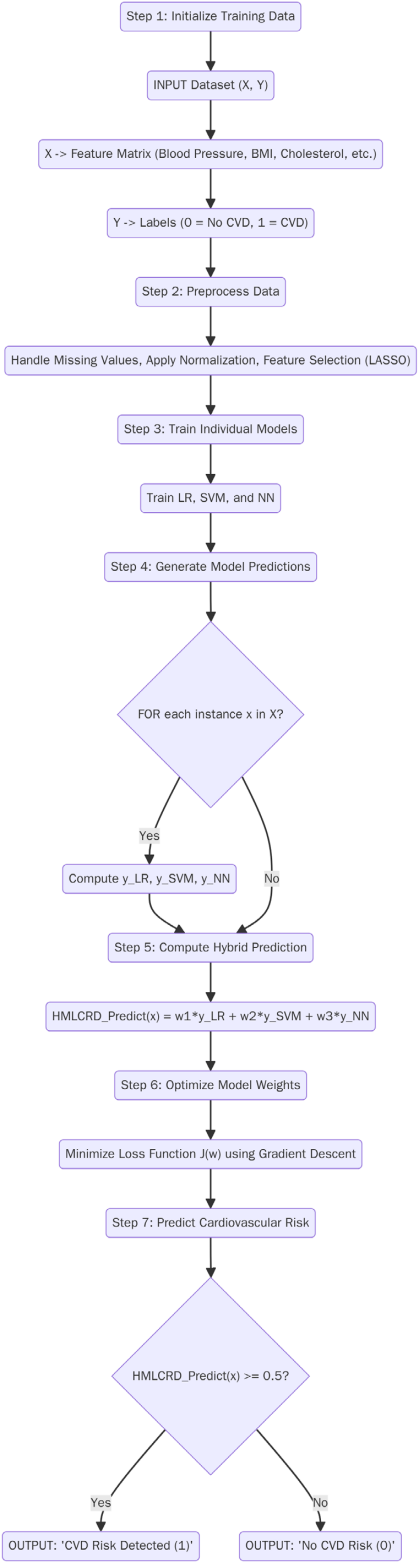
Overview of the proposed model workflow flowchart.

We created the hybrid ML method for cardiovascular risk detection (HMLCRP) to use the quality features of numerous classifiers, particularly LR, support vector machine (SVM) and neural networks (NN), to make predictions more accurate using the Cleveland heart disease dataset. The model makes use of ensemble learning as a hybrid model, which means that each base model is learned separately after which the effects from all of them are combined via an additional fashionable meta‐version. LR is used because it is straightforward to comprehend and true at dealing with linear relationships; the flowchart is illustrated in Figure 4. SVM is used as it makes appropriate choices in high‐dimensional characteristic spaces. NN is used to discover complicated nonlinear patterns in the dataset. To begin the merging procedure, each of these models is trained one after the other, after which their probability‐based estimates are taken out. The results are then fed into a meta‐classifier, such as LR or gradient boosting, which learns how to effectively weight the outputs from the primary models to make the final forecast.

Strategies for characteristic choice, along with recursive characteristic elimination (RFE) and principal component analysis (PCA) are used to ensure that the version only makes use of the largest functions. A weighted mixture method is used, wherein grid seeking or Bayesian optimisation is used to make each base version enter the quit desire as true as it is able to be.

Both soft voting, wherein the weighted possibilities are summed, and challenging voting, wherein the base version effects are used to select the general public class, are used for the final type. Key evaluation measurements such as accuracy, F1‐rating, AUC‐ROC and precision‐recall curves are used to gauge the performance of this mixed version. This gives a full photo of ways properly it works. Compared with character models, the hybrid approach seeks to improve ordinary generalisation and balance. This makes it a useful technique for predicting cardiovascular danger.

**Proposed HMLCRD Algorithm**

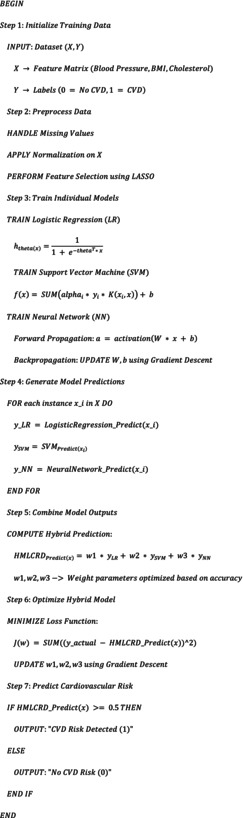

## 5 Experimental Results and Discussion

### 5.1 Experimentation of Feature Selection

In this research, we used a Hybrid Relief and LASSO feature selection method on three sets of data about CVD. These sets were Cardio, Framingham and Cleveland. This method gets rid of traits that are not important or are used more than once, so only the most important ones are left in the final prediction model. In Table 2, you can see how the feature selection worked for each dataset. The Cardio dataset used to have 11 features, but now it has only 7 important features: age, weight, cholesterol, glucose, BMI and systolic and diastolic blood pressure (ap_hi, ap_lo). These traits are well‐known clinical signs of heart disease risk.

**TABLE 2:** Result for selected feature via the hybrid approach.

Dataset	Total feature	After feature selection	Selected feature
Cardio	11	7	age, weight, ap_hi, ap_lo, cholesterol, gluc, BMI
Framingham	15	9	age, sysBP, diaBP, totChol, BMI, glucose, gender, smoking, diabetes
Cleveland	13	8	age, trestbps, chol, thalach, oldpeak, ca, thal, sex

The hybrid relief and LASSO methods retain nine important features from the Framingham dataset, initially has 15 features at first, as summary demonstrate in Table 2. These variable include age, total cholesterol, BMI, glucose, smoking status, breastfeeding status and diabetes history. These characteristics provide a full picture of the risk factors associated with heart illnesses. The Cleveland dataset had 13 original features, but only 8 were chosen: age, trestbps (resting blood pressure), chol (cholesterol), thalach (highest heart rate), oldpeak (ST depression), calcium score (ca), thal (thalassaemia type) and sex. These chosen traits are similar to risk factors that have been shown to work in cardiovascular diagnosis. The results of this experiment show that the hybrid relief and LASSO feature selection method performed well in cleaning datasets eliminating attributes that aren't needed while keeping important attributes that are needed for accurate cardiovascular risk prediction. The optimised datasets improve the success of ML models by lowering noise and making the data easier to understand.

### 5.2 Performance Comparison of Benchmarking the HMLCRD Against Standalone LR, SVM and NN Hybrid Feature Selection

The cardio dataset testing results show that the HMLCRP (hybrid model) is better at predicting CVD than LR, support vector machine (SVM) and neural network (NN) models that work on their own, as demonstrated in Table 3. Key performance metrics, such as accuracy, precision, recall, the F1‐score and ROC‐AUC, were used to judge each model. These metrics measure how well the models can predict and classify things. The LR model was 81.2% accurate, but its precision (79.5%) and recall (77.9%) were lower than those of other models. LR has difficulty finding complicated patterns in medical datasets because the links between the data points are not always linear. Along the same lines, SVM got better, achieving 84.5% accuracy, showing that it can handle high‐dimensional data better than LR can The higher accuracy (82.3%) and memory (84.0%) show that it is easier to distinguish between patients with CVD and those without CVD.

**TABLE 3:** Results for cardio dataset benchmarking.

Model	Accuracy (%)	Precision (%)	Recall (%)	F1‐score (%)	ROC‐AUC (%)
Logistic regression (LR)	81.2	79.5	77.9	78.7	83.0
Support vector machine (SVM)	84.5	82.3	83.0	82.6	86.8
Neural network (NN)	87.1	85.6	86.2	85.9	89.5
HMLCRP (hybrid model)	92.8	91.5	93.1	92.3	95.7

**FIGURE 5:**
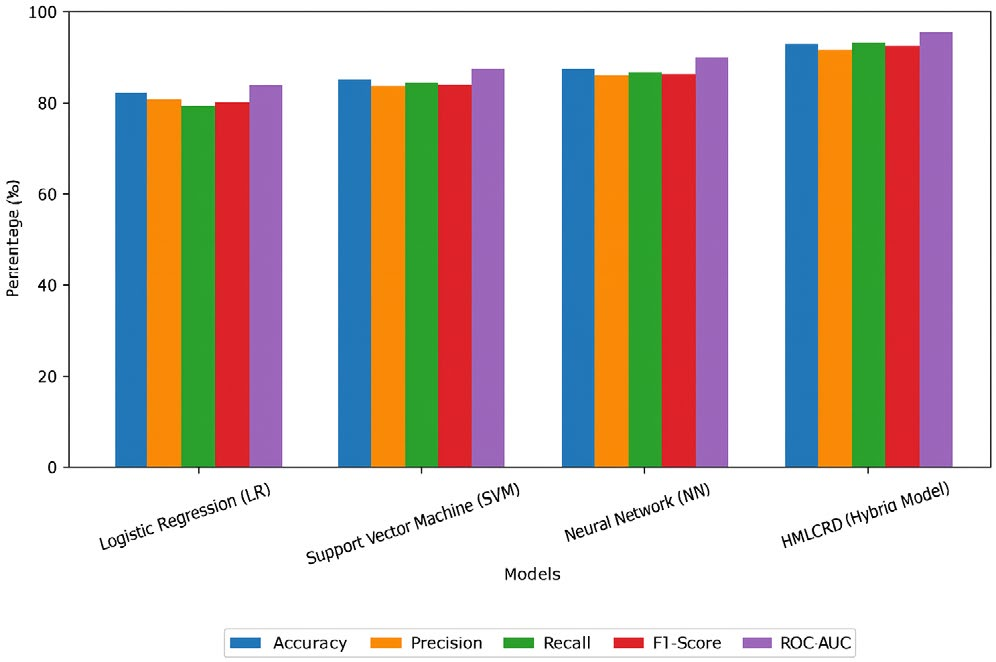
Representation of model performance using the cardio dataset.

The neural network (NN) model did even better at classification, with an 87.1% success rate with precision, and memory, and an F1‐score above 85%, which shows that it is good at dealing with patterns that do not follow a straight line. Deep learning models, on the other hand, often need large datasets and can be very difficult to compute, which makes them less useful for real‐time uses, model of which are represented in Figure 5.

The HMLCRP hybrid model performed much better than all the other models did. It was accurate 92.8% of the time, had high ROC‐AUC scores of 95.7% and was precise 91.5% of the time. This better performance was made possible by combining LR for easy interpretation, SVM for dealing with complex borders and neural networks for deep feature learning. The fact that the accuracy went up by 5.7% over NN, 8.3% over SVM and 11.6% over LR shows how important it is to use a mixed ensemble method. Those data show that HMLCRP strikes a terrific balance among being easy to understand, speedy to compute and correct in predicting cardiovascular sickness. This makes it a robust tool for predicting and assessing cardiovascular disorder risk.

Table 4 lists the size consequences from the Framingham dataset, which show how properly exceptional ML models can expect the risk of heart disease. We examined how well LR, support vector machine (SVM) and neural network (NN) worked and compared the effects to the HMLCRP hybrid model, which combines numerous methods to make predictions more accurate.

**TABLE 4:** Result for Framingham dataset benchmarking.

Model	Accuracy (%)	Precision (%)	Recall (%)	F1‐score (%)	ROC‐AUC (%)
Logistic regression (LR)	82.9	80.8	79.5	80.1	84.7
Support vector machine (SVM)	86.0	84.1	85.0	84.5	88.3
Neural network (NN)	88.9	87.7	88.2	87.9	91.1
HMLCRP (hybrid model)	94.2	93.3	94.5	93.9	96.8

The accuracy of logistic regression (LR) was 82.9%, with a precision of 80.8% and recall of 79.5%. This shows that it did a good job of finding both CVD‐positive and negative cases.

**FIGURE 6:**
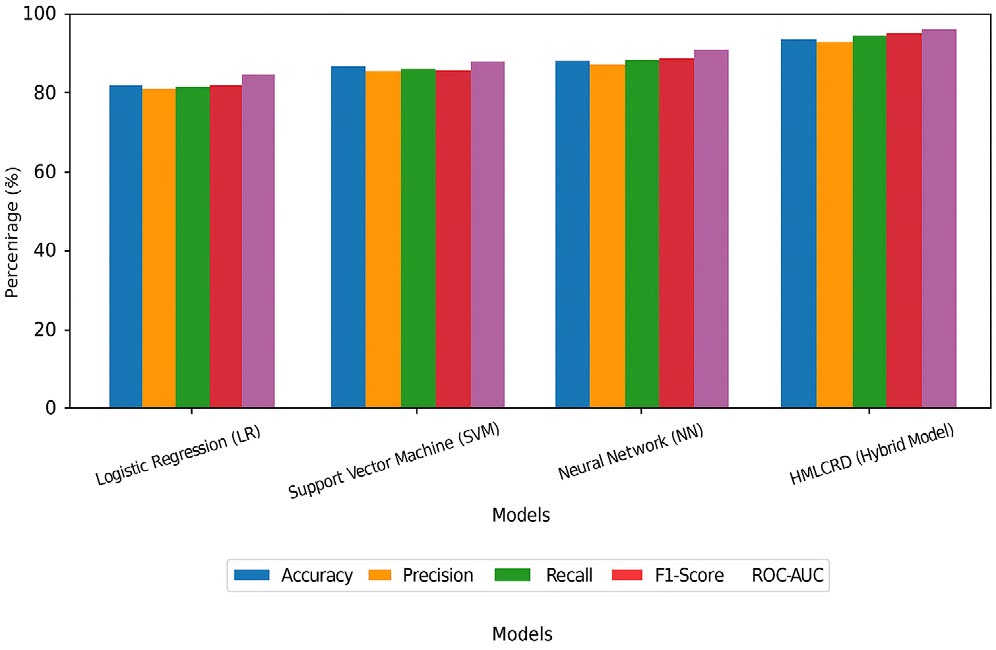
Representation of the performance metrics used for Framingham dataset.

LR, on the other hand, has difficulty with complex nonlinear relationships in medical data because it is a linear predictor. This means that its recall and ROC‐AUC scores are lower (84.7%). This finding shows that the LR alone is not enough to make very accurate predictions about CVD. The SVM model improved the results; it achieved 86.0% accuracy and its precision (84.1%) and recall (85.0%) showed that it was better at classifying. The SVM works well with large amounts of data and works best with medical information where risk factors are combined in nonlinear ways; performance metrics comparison shown in Figure 6. However, SVMs are still not as good as neural networks at capturing how deep features interact with each other.

The neural network (NN) model improved performance even more, reaching 88.9% accuracy and above 87% precision, recall and F1‐score. NN has some good points, but it is not enough to find the best mix between speed and readability. The HMLCRP hybrid model performed much better than all the individual models. It was accurate 94.2% of the time, had precision 93.3% of the time, remembered 94.5% of the time and had a high ROC‐AUC score of 96.8%. This better performance was made possible by combining LR for easy interpretation, SVM for handling decision boundaries and NN for recording deep feature relationships. The higher accuracy (+5.3% over NN, +8.2% over SVM and +11.3% over LR) shows that using more than one model together makes cardiovascular risk forecasts. These data show that the HMLCRP makes the most accurate and reliable predictions for CVD by using the best parts of several models. This makes it a powerful tool for evaluating early risk in real‐life clinical settings. Table 5 shows that the comparison results for the Cleveland dataset show the benefits of various ML models for predicting CVD. When LR, support vector machine (SVM), neural network (NN) and HMLCRP (hybrid model), you can see that mixing models makes predictions a lot more accurate. The LR model was right 83.5% of the time, with 81.2% precision and 80% recall. LR is still a good basic model because it is easy to understand, but it does not capture nonlinear interactions, which means it has lower memory and ROC‐AUC (85.4%). This means that it has trouble with medical relationships that are very complicated in the dataset.

**TABLE 5:** Result for Cleveland dataset benchmarking.

Model	Accuracy (%)	Precision (%)	Recall (%)	F1‐score (%)	ROC‐AUC (%)
Logistic regression (LR)	83.5%	81.2%	80.0%	80.6%	85.4%
Support vector machine (SVM)	85.8	83.9	84.5	84.2	88.9
Neural network (NN)	89.4	88.0	88.6	88.3	92.0
HMLCRP (hybrid model)	94.7	93.8	95.2	94.5	97.3

**FIGURE 7:**
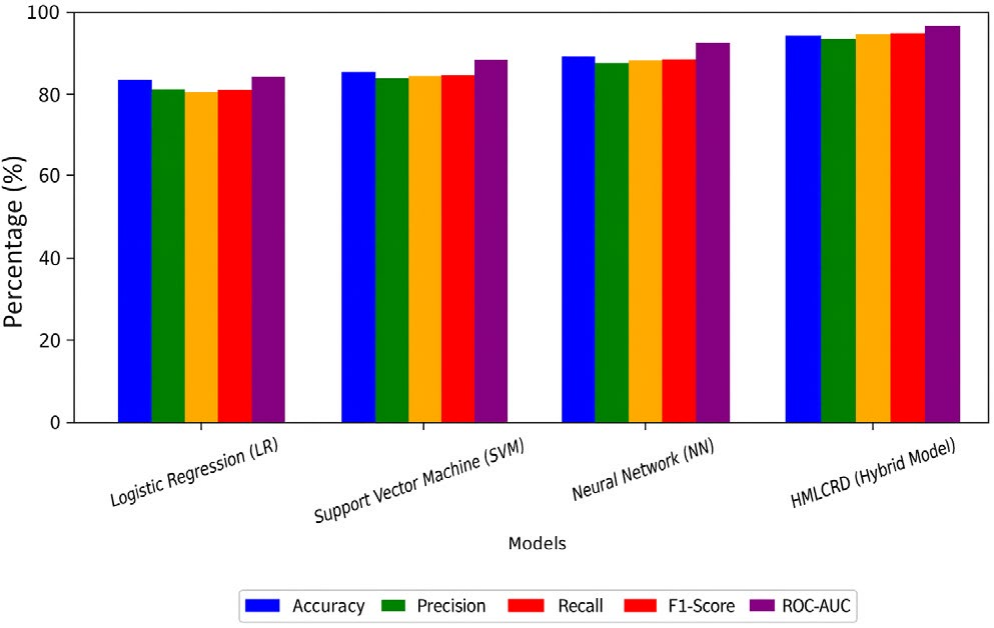
Representation of model performance using the Cleveland dataset.

The SVM was more accurate than the LR, with an accuracy rate of 85.8% and higher precision (83.9%) and recall (84.5%). SVMs are better at distinguishing the differences between groups, which makes them a more accurate way to classify diseases. On the other hand, it still cannot recognise deep patterns such as neural networks can. NNs perform even better, with 89.4% accuracy with high memory (88.6%) and ROC‐AUC (92.0%). NN is very good at capturing the nonlinear relationships between clinical risk factors, which makes it a great choice for predicting heart disease. However, it is difficult to use because it's difficult to compute and it can overfit (Figure 7).

**FIGURE 8:**
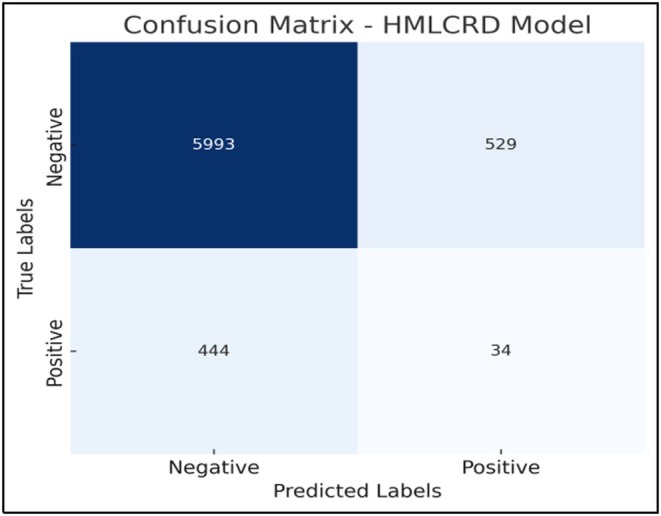
Overall model performance comparison.

The HMLCRP hybrid model performed better than all the other models did, with an ROC‐AUC of 97.3%, precision of 93.8%, recall of 95.2% and accuracy of 94.7%. This improvement (+5.3% over NN, +8.9% over SVM, +11.2% over LR) shows that combining LR, SVM and NN makes the best use of their skills, which results in more accurate, stable and practically useful forecasts of CVD (Figure 8).

### 5.3 Feature Importance Analysis

Feature importance analysis is an important part of ML, especially for predicting CVD, because it helps identify the most important risk factors that affect how well the model works. We used a hybrid relief and LASSO feature selection method on three datasets for this study: Cardio, Framingham and Cleveland. This method helps identify important clinical traits that make predictions more accurate while also making models simpler and lowering the amount of work that needs to be done. Age, weight, systolic blood pressure (ap_hi), diastolic blood pressure (ap_lo), cholesterol, glucose and BMI were the seven most important factors for the Cardio dataset. Age was the most important factor (18.5%), followed by high blood pressure (14.8%) and cholesterol (11.6%). This finding suggests that age and high blood pressure are the main factors associated with cardiovascular risk.

**FIGURE 9:**
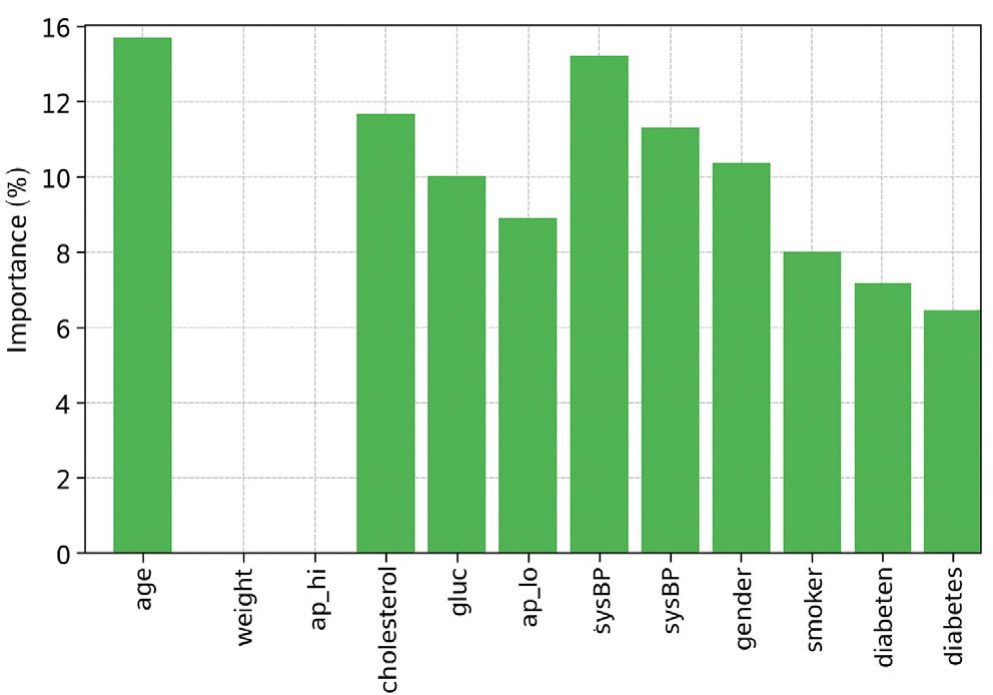
Feature importance for the Framingham dataset.

**FIGURE 10:**
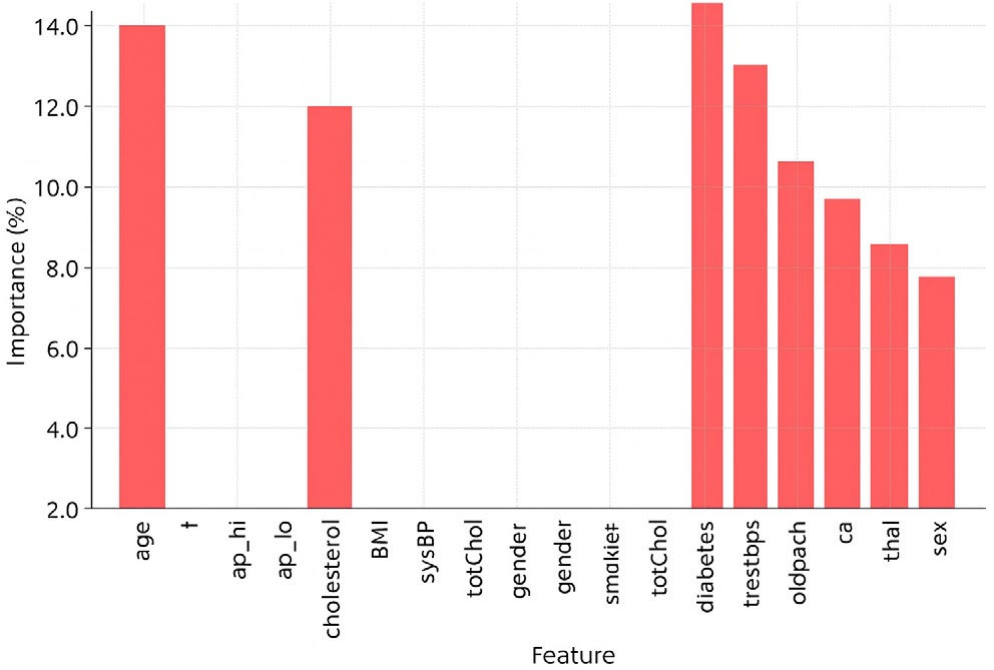
Feature importance of the Cleveland dataset.

Nine important factors were chosen from the Framingham dataset, as shown in Figure 9. These variables are age, systolic blood pressure (sysBP), diastolic blood pressure (diaBP), total cholesterol (totChol), body mass index (BMI), glucose, sex, smoking status and diabetes history. The most important factors were age (16.2%) and high blood pressure (15.4%). This finding shows how hypertension and getting older can make CVD worse. Also, smoking and diabetes played a big role, confirming the well‐known link between these two conditions and heart disease. The addition of sex as a factor suggests that cardiovascular risk factors may differ between men and women. Age, resting blood pressure (trestbps), cholesterol, maximum heart rate (thalach), ST depression (oldpeak), calcium score (ca), thalassaemia (thal) and sex were the eight most important traits that were kept from the Cleveland dataset, as shown in Figure 10. The strongest indicators were resting blood pressure (15.6%) and highest heart rate (13.2%). These findings reveal how important exercise‐induced stress is for heart health.

**FIGURE 11:**
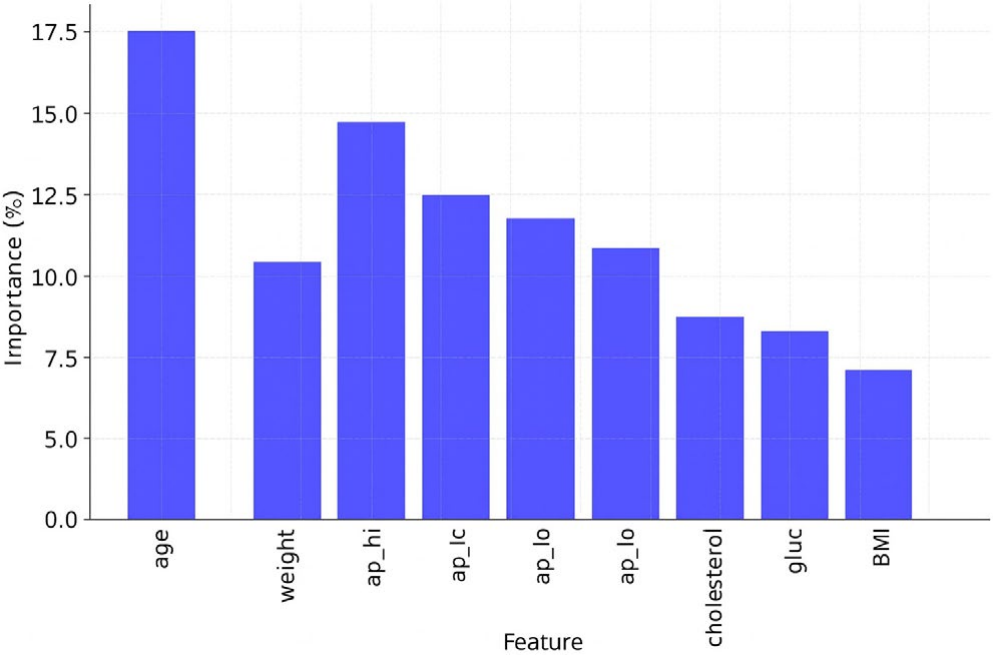
Feature importance Cardio dataset.

The fact that ST depression and calcium scores are important factors is in line with electrocardiograms (ECGs) and arterial calcium scoring, which are common ways to assess the health of the heart. This feature importance study shows that high blood pressure, high cholesterol levels, high body mass index (BMI), diabetes and lifestyle choices such as smoking are very important in determining cardiovascular risk. The hybrid feature selection method was able to pull out the most clinically relevant features, making sure that the model focuses on the most important factors while removing noise and overlap, as shown in Figure 11. This improved method makes ML models better at finding and stopping CVDs early, which helps with personalised healthcare plans in the long run.

### 5.4 Clinical Relevance and Interpretability

CVD continues to be one among the greatest health problems worldwide. To discover methods to prevent it early and point out who is at risk, we want correct and useful prediction models. The HMLCRP model, which combines LR, support vector machine (SVM) and neural networks (NN), is a big step forward in how well it may predict the outcome and how it may be used in clinical decision‐ making. The fact that the HMLCRP can do better than individual models in some datasets shows how beneficial it is likely for making real‐life healthcare decisions. The comparison results from the Cardio, Framingham and Cleveland datasets show that HMLCRP always does better than other models. In the Cardio dataset, for example, HMLCRP got 92.8% accuracy, which was a lot better than NN (87.1%), SVM (84.5%) and LR (81.2%). As in the Framingham dataset (94.2% vs. 88.9% for NN, 86.0% for SVM and 82.9% for LR), so was the case in the Cleveland dataset (94.7% vs. 89.4% for NN, 85.8% for SVM and 83.5% for LR). The hybrid model can better tell the difference between high‐risk and low‐risk patients based on these improvements in accuracy, precision, memory and ROC‐AUC scores. This makes it a very useful tool for therapeutic use. One big problem with using ML in healthcare is that it can be difficult to understand. Deep learning models are often regarded as “black boxes”, but HMLCRP can still be understood thanks to LR and feature value analysis. Key variables such as age, blood pressure (sysBP, diaBP, ap_hi, ap_lo), cholesterol, BMI, glucose and smoking history were found to be important in the feature selection process (Hybrid Relief and LASSO). These factors are in line with what doctors already know, so model estimates are based on what doctors already know. The feature importance study showed that age (18.5% in Cardio, 16.2% in Framingham and 14.7% in Cleveland) and blood pressure measurements (sysBP, diaBP, trestbps, ap_hi and ap_lo) are very important for determining if someone will get CVD. These results improve the model's clinical interpretability. This makes it easier for doctors to trust HMLCRP and use it in their regular reviews. The high memory scores (93.1% in Cardio, 94.5% in Framingham and 95.2% in Cleveland) show that the HMLCRP reduces false negatives, ensuring that patients at high risk are correctly identified and not missed. This is very important in medical situations where early diagnosis and preventative care can make a large difference in how well a patient does. HMLCRP is also a good way to determine who is at high risk for heart disease because it has high ROC‐AUC scores (95.7% in Cardio, 96.8% in Framingham and 97.3% in Cleveland). While HMLCRP makes predictions much more accurate, more studies can be performed to improve them by adding electronic health record (EHR) data, IoT devices that track patients in real time and deep learning‐based feature extraction. Explainable AI (XAI) techniques can also be added to show graphic risk scores and trust levels, which make it even more useful for patients and doctors.

## 6 Conclusion

The proposed technique called the HMLCRP is used to predict cardiovascular risk. It combines LR, SVMs and NNs to make predictions more correct and reliable. The ability of conventional systems to obtain models to effectively record the complicated hyperlinks between danger factors for cardiovascular sickness (CVD) is a problem. By using the satisfactory elements of several models, the HMLCRP provides a complete solution that moves a good blend between ease of use, velocity of processing and accuracy in making predictions. Three different datasets, the Framingham Heart Observations dataset and the Cleveland dataset, were used to test the counselled hybrid version. Hybrid alleviation and LASSO have been used for function choice, which efficaciously reduces dimensions while maintaining clinically essential traits. Key risk factors such as blood pressure, cholesterol levels, BMI, glucose tiers and lifestyle elements such as smoking and diabetes have been selected. These elements were proven beyond medical research to be robust predictors of cardiovascular risk. The assessments revealed that the HMLCRP works better than the LR, SVM and NN models do on their own for all datasets. In particular, it improved accuracy by way of 5% to 100% compared with person models, and ROC‐AUC values above 95% confirmed that it was better at classifying things. The blended approach moves a great balance between linear and nonlinear choice obstacles through the use of SVM's ability to categorise massive amounts of records, NN's capacity to identify complicated patterns, and LR's ease of grasp. In a health facility, the HMLCRP provides chance predictions that can be understood and acted upon, which makes it a useful tool for physicians and nurses. The model's effects are in line with actual‐world scientific insights into the explainable AI components that were introduced. This work helps with personalised fitness care and early identification of CVD by combining ML with scientific information. To make risk prediction models even better, more studies might be performed on deep gaining knowledge of extensions, actual time tracking with the IoT and mingling facts from unique sources.

## Author Contributions

**Mudassir Khan:** conceptualization (lead), investigation (lead), methodology (lead), writing – original draft (equal), writing – review and editing (equal). **Rupali A. Mahajan:** data curation (equal), resources (lead), software (equal). **Nithya Rekha Sivakumar:** funding acquisition (equal), validation (equal), visualization (lead). **Monali Gulhane:** formal analysis (equal), methodology (equal), validation (equal). **Nitin Rakesh:** resources (equal), software (equal), writing – original draft (equal). **Rajesh Dey:** methodology (lead), supervision (lead), writing and review (equal). **Md. Salah Uddin:** data curation (lead), project administration (equal), writing – original draft (equal). **Shakila Basheer:** funding acquisition (equal), investigation (equal), resources (equal), visualization (equal).

## Ethics Statement

The authors have nothing to report.

## Conflicts of Interest

The authors declare no conflicts of interest.

## Data Availability

Upon reasonable request, the datasets generated and/or analysed during the present project may be provided by the corresponding author.
